# Supplementary material for: Capturing human categorization of natural images by combining deep networks and cognitive models
Source: Nat Commun. 2020 Oct 27;11:5418. doi: 10.1038/s41467-020-18946-z (PMC7591513; doi:10.1038/s41467-020-18946-z)
Supplement: Supplementary file 2 — Reporting Summary [file 41467_2020_18946_MOESM2_ESM.pdf]

nature research

Corresponding author(s): Ruairidh M. Battleday
Joshua C. Peterson

Last updated by author(s): Sep 1, 2020

Reporting Summary

Nature Research wishes to improve the reproducibility of the work that we publish. This form provides structure for consistency and transparency in reporting. For further information on Nature Research policies, see our Editorial Policies and the Editorial Policy Checklist.

Statistics

For all statistical analyses, confirm that the following items are present in the figure legend, table legend, main text, or Methods section.

n/a	Confirmed	
☐	☒	The exact sample size (*n*) for each experimental group/condition, given as a discrete number and unit of measurement
☐	☒	A statement on whether measurements were taken from distinct samples or whether the same sample was measured repeatedly
☒	☐	The statistical test(s) used AND whether they are one- or two-sided<br>*Only common tests should be described solely by name; describe more complex techniques in the Methods section.*
☐	☒	A description of all covariates tested
☐	☒	A description of any assumptions or corrections, such as tests of normality and adjustment for multiple comparisons
☒	☐	A full description of the statistical parameters including central tendency (e.g. means) or other basic estimates (e.g. regression coefficient) AND variation (e.g. standard deviation) or associated estimates of uncertainty (e.g. confidence intervals)
☒	☐	For null hypothesis testing, the test statistic (e.g. *F*, *t*, *r*) with confidence intervals, effect sizes, degrees of freedom and *P* value noted<br>*Give P values as exact values whenever suitable.*
☐	☒	For Bayesian analysis, information on the choice of priors and Markov chain Monte Carlo settings
☒	☐	For hierarchical and complex designs, identification of the appropriate level for tests and full reporting of outcomes
☒	☐	Estimates of effect sizes (e.g. Cohen's *d*, Pearson's *r*), indicating how they were calculated

Our web collection on statistics for biologists contains articles on many of the points above.

Software and code

Policy information about availability of computer code

Data collection	Javascript libraries: jspsych<br>nodesjs libraries: body-parser, ejs, express, mongoose<br>Our experiment was an extensive adaptation of the repo here: https://github.com/Tuuleh/jsPsychBackendStart
Data analysis	python version 3.6.5 was used for the data analysis, with numpy package version 1.15.1.<br>The python package Theano version 0.8.2 was used for model training and optimization.<br>The python packages scipy version 1.0.1 and matplotlib version 2.1.1 were used for the simulations and data presentation.<br>Caffe version 1.0 was used to generate the AlexNet representations.<br>Keras version 1.10 was used to generate the DenseNet representations.

For manuscripts utilizing custom algorithms or software that are central to the research but not yet described in published literature, software must be made available to editors and reviewers. We strongly encourage code deposition in a community repository (e.g. GitHub). See the Nature Research guidelines for submitting code & software for further information.

Data

Policy information about availability of data

All manuscripts must include a data availability statement. This statement should provide the following information, where applicable:

- Accession codes, unique identifiers, or web links for publicly available datasets
- A list of figures that have associated raw data
- A description of any restrictions on data availability

The dataset presented in the current work (CIFAR-10H) is available in a public GitHub repository: https://github.com/jcpeterson/cifar-10h (DOI: 10.5281/

zenodo.4008585). The image dataset (CIFAR-10) can be found at https://www.cs.toronto.edu/~kriz/cifar.html.

Field-specific reporting

Please select the one below that is the best fit for your research. If you are not sure, read the appropriate sections before making your selection.

☐ Life sciences ☒ Behavioural & social sciences ☐ Ecological, evolutionary & environmental sciences

For a reference copy of the document with all sections, see nature.com/documents/nr-reporting-summary-flat.pdf

Behavioural & social sciences study design

All studies must disclose on these points even when the disclosure is negative.

Study description	This is a quantitative cognitive science study investigating human categorization behaviour. We collected a dataset of human categorizations of natural images in an online experiment using Amazon Mechanical Turk, and then analyzed their distributions using a number of computational models of categorization, and machine learning representations of images. The goal was to assay the contributions and interplay of different image representations and different categorization strategies in building successful models of human categorization behaviour.
Research sample	Our participants were Amazon Mechanical Turk (AMT) workers from the United States. Selection criteria was 98%+ approval rate. 2570 participants completed the task. Although we did not explicitly collect demographic information. General statistics about the characteristics of AMT workers can be found in: <br>Ross, J., Zaldivar, A., Irani, L., & Tomlinson, B. (2009). Who are the turkers? Worker demographics in Amazon Mechanical Turk. Department of Informatics, University of California, Irvine, USA, Tech. Rep.
Sampling strategy	50 judgments were collected for each image, based on an assessment of the number of judgments required to provide clear resolution of the distribution of categories for each image.
Data collection	Data was collected on AMT, and so all participants completed the experiment from a remote computer, using only a mouse to provide responses. Participants saw an image presented centrally on their screen, and had to click one of ten surrounding buttons, each labelled with a different category, with their computer mouse. There was an initial training phase, during which participants had to score at least 75% accuracy, split into 3 blocks of 20 images taken from the CIFAR-10 training set (60 total, 6 per category). If participants failed any practice block they were asked to redo it until passing the threshold accuracy. After successful practice, each participant categorized 200 images selected at random (20 from each category) for the main experiment phase. After an image had received 50 or more categorizations, it was removed from the pool. After every 20 trials, there was an attention check trial using a carefully selected unambiguous member of a particular category taken from the CIFAR-10 training set. There were no contrastive experimental conditions or hypotheses to blind researchers to.
Timing	Began 03/03/18 and ended 03/25/18.
Data exclusions	Data for 14 participants was excluded, based on performance beneath a pre-established threshold (75%) on a set of attention checks during main data collection.
Non-participation	No participants dropped out or declined participation.
Randomization	No between-groups comparison in this study that required randomization.

Reporting for specific materials, systems and methods

We require information from authors about some types of materials, experimental systems and methods used in many studies. Here, indicate whether each material, system or method listed is relevant to your study. If you are not sure if a list item applies to your research, read the appropriate section before selecting a response.

Materials & experimental systems

n/a	Involved in the study	
☒	☐	Antibodies
☒	☐	Eukaryotic cell lines
☒	☐	Palaeontology and archaeology
☒	☐	Animals and other organisms
☐	☒	Human research participants
☒	☐	Clinical data
☒	☐	Dual use research of concern

Methods

n/a	Involved in the study	
☒	☐	ChIP-seq
☒	☐	Flow cytometry
☒	☐	MRI-based neuroimaging

April 2020

Human research participants

Policy information about studies involving human research participants

Population characteristics	See above.
Recruitment	Participants were recruited through AMT, and as such are subject to a range of self-selection and selection biases that are more fully detailed elsewhere. None of these were a direct result of the present study, and we unlikely to affect our analysis of basic categorization behavior.
Ethics oversight	The dataset was collected while the authors were at UC Berkeley under IRB approval, protocol: Cognitive Research Using Amazon Mechanical Turk (Expedited) -- 2015-05-7551. All participants gave informed consent.

Note that full information on the approval of the study protocol must also be provided in the manuscript.
